# Heart rate analysis in neonatal sepsis: a complex equation

**DOI:** 10.1038/s41390-024-03548-y

**Published:** 2024-09-06

**Authors:** Brynne A. Sullivan, Karen D. Fairchild

**Affiliations:** Hospital Drive, University of Virginia School of Medicine, Charlottesville, VA 22908 USA

Understanding the pathophysiology of sepsis will allow clinicians to identify and treat patients in an earlier (non-obvious) stage of illness, which is likely to improve outcomes. Neonates are unique not only in their immature host defense mechanisms leading to increased risk for sepsis but also in their immature autonomic nervous system and other physiologic processes regulating vital signs. Since heart rate is routinely, and often continuously, monitored in hospitalized neonates, it is a good physiologic marker on which to focus when developing sepsis early warning systems.

The article by Pedersen et al. “Lipopolysaccharide-induced systemic inflammation and heart rate variability in a term newborn piglet model,”^1^ describes changes in vital signs and laboratory values in piglets on the day of birth during an intravenous infusion of E. coli LPS. Animal experiments are important for understanding sepsis physiology, and the authors should be commended for following the “RRR” ethical principles of animal research (replacement, refinement, reduction),^2^ by reporting this as a substudy of a larger evaluation of neonatal sepsis physiology in the same animals. The major finding was that a substantial increase in heart rate during endotoxin infusion mirrored a decline in heart rate variability (HRV). Changes in other vital signs and laboratory values were described, validating their model as one that mimics many aspects of Gram-negative early-onset neonatal sepsis.

Tachycardia is a well-described response to endotoxemia, reflecting sympathetic nervous system activation and consequences of a robust systemic inflammatory response. In the Pedersen study, the rise in heart rate was not seen until 2 h into the LPS infusion, reflecting the time required for inflammatory cytokines to be produced and to cause endothelial cell damage, capillary leak, hypovolemia, and hypotension which was temporally associated with the rise in heart rate. Human studies have yielded similar findings. In the 1990’s, Suffredini et. al. conducted studies at the National Institutes of Health Clinical Center in healthy adult volunteers, in which tachycardia was noted 2–3 h after starting a continuous endotoxin infusion.^3^

Higher heart rate *ipso facto* leads to decreased variability of inter-heartbeat (RR) intervals. HRV is classically measured from time series data as “SDNN” (standard deviation of normal-to-normal heartbeats), and the Pedersen group appropriately use this metric and others, including a non-linear one (Poincare plot) and frequency-based power spectrum analysis, reflecting sympathetic and parasympathetic nervous system activation (low and high-frequency HRV, respectively). All HRV measures declined during the endotoxin infusion, but multivariable logistic regression revealed that the change in HRV was entirely due to the increase in heart rate. While this is an informative finding, there are several important caveats. First, depressed HRV can occur as a sign of pathology with or without a significant change in HR. Second, healthy infants can have tachycardia with normal HRV. Third, there are HRV measures that are less impacted by HR, such as approximate entropy.^3^ And finally, concluding that monitoring HR may be as good as monitoring HRV for detecting neonatal sepsis is simplifying a complex problem.

A particularly important consideration regarding HR analysis for early detection of neonatal sepsis is gestational age. Preterm infants, who constitute about 10% of all births and are at more than 10-fold higher risk for sepsis than term infants, have higher baseline HR and are prone to occurrence of HR decelerations in part due to immature control of breathing. A common presenting sign of sepsis in preterm infants is increased episodes of central apnea, which are often accompanied by bradycardia. Even in absence of apnea (for example while on mechanical ventilation), preterm infants with sepsis often have repetitive transient decelerations of HR, which are at least in part due to vagus nerve firing. In a mouse model, we showed that intraperitoneal injection of Gram-negative or Gram-positive bacteria or Candida albicans induced repetitive decelerations which were associated with activation of vagal centers in the brainstem, and which were blocked by atropine,^4^ likely representing activation of a cholinergic anti-inflammatory pathway. This is a critical host defense mechanism whereby, in response to pathogen invasion, vagus nerve firing leads to acetylcholine release which, via nicotinic cholinergic receptors on macrophages, suppresses production of pro-inflammatory cytokines.^5^ This important counterregulatory mechanism serves to avoid a tissue-damaging and potentially fatal “cytokine storm”.

**Fig. 1:**
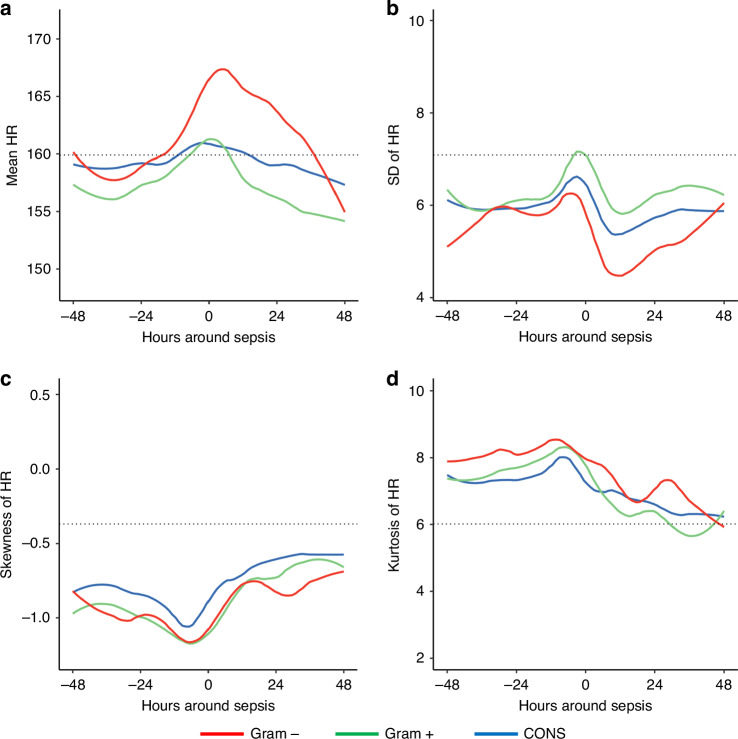
Four mathematical moments of heart rate in preterm late-onset sepsis. HR mean (**a**), standard deviation (**b**), skewness (**c**) and kurtosis (**d**) are shown in the 96 h period before and after 301 episodes of blood culture-positive sepsis (at time zero) in very low birthweight infants at 4 Level 4 NICUs (mean gestational age 25.6 weeks). HR characteristics are shown for sepsis with Gram-negative bacteria (*n* = 56), CONS (*n* = 195) and other Gram-positives (*n* = 50). The horizontal dotted line is the mean value of each HR characteristic for all VLBW infants at all times during the NICU stay.

One signature of sepsis in premature infants is decreased HRV that is punctuated by transient HR decelerations. This pattern is very familiar to obstetricians, since fetal distress (sometimes due to chorioamnionitis and a fetal inflammatory response) manifests a similar pattern. Decelerations confound use of low HRV as a sepsis biomarker, and this paradox was addressed when developing the Heart Rate Characteristics Index, or HeRO Score, as an early warning system for sepsis in premature infants. The algorithm incorporates a measure of low HRV, a measure of decelerations (sample asymmetry), and sample entropy, which reflects irregularity of HR. The HeRO score represents the fold increased risk that, within the next 24 h, a preterm infant will be diagnosed with sepsis.^6^ In a randomized trial of 3003 preterm very low birth weight infants at 9 Neonatal Intensive Care Units, display of this score reduced sepsis-associated mortality from 20 to 12%.^7^ Of note, in the HeRO study, mean HR only increased slightly in the hours near sepsis diagnosis, from 159 to 160 beats per minute, whereas the mean HeRO score rose in a readily visible manner.^8^ Figure 1 depicts more recent data from 301 episodes of blood culture-positive sepsis in very low birthweight infants at four NICUs, with four mathematical moments of heart rate shown at the time of the positive blood culture (time zero) and 48 h before and after. Panel A shows that mean HR increased more during sepsis caused by Gram-negative organisms (*n* = 56) than sepsis caused by Coagulase-negative staphylococcus (CONS, *n* = 195) or other Gram-positive organisms (*n* = 50). Panel B shows that HR standard deviation (SD) rose slightly before diagnosis, and this was mirrored by more negative skewness (Panel C), indicating more decelerations. After diagnosis (during antibiotic treatment), HR SD declined more for Gram-negative than for Gram-positive pathogens. Panel D shows the fourth mathematical moment, kurtosis, which reflects the shape of the HR histogram tails. The rise in kurtosis indicates the widened distribution of HR with more high and low values.

Monitoring HR (and indeed all vital signs) is a cornerstone of clinical care for neonates at risk for sepsis. Pederson et. al. raise the important point that HRV analysis is not currently available in the vast majority of clinical settings, and clinicians must examine raw vital signs for things like tachycardia as a tip-off to a systemic inflammatory response. More complex analyses will capture nuances of neonatal pathophysiology and may detect changes even earlier in the course of illness. The HeRO monitor is the first artificial intelligence (AI)-based early warning system for neonatal sepsis prediction, but others are in the research pipeline. Next-generation algorithms will incorporate other continuously monitored physiologic data such as oxygen saturation, which can inform on respiratory distress and apnea.^9,10^ Since HR and SpO2 instability may occur in preterm infants without sepsis, and since overuse of antibiotics is detrimental, it is critical to consider all available clinical and laboratory data in making decisions about whether to obtain cultures and start treatment, or wait and watch closely.

AI will be an important addition to neonatal care, but its impact is likely to be greatest if the technology is explainable, trusted, and properly implemented.^11^ Even more importantly, new AI should be tested in randomized clinical trials to assess impact on important outcomes, so that we are not simply adding more data to clinicians’ already data-filled days, but alerting them to a physiologic change that warrants a bedside evaluation.
